# Relationships between antibiotic exposure and asthma in adults in the United States: results of the National Health and Nutrition Examination Survey between 1999 and 2018

**DOI:** 10.3389/fpubh.2023.1123555

**Published:** 2023-04-27

**Authors:** Shaoli Li, Feilong Chen, Chunlei Huang, Guimin Huang, Yijing Cheng, Tao Li, Dongqing Hou, Wenqian Liu, Tao Xu, Junting Liu

**Affiliations:** ^1^Child Health Big Data Research Center, Capital Institute of Pediatrics, Beijing, China; ^2^Department of Epidemiology and Statistics, Institute of Basic Medical Science, Chinese Academy of Medical Sciences & School of Basic Medicine, Peking Union Medical College, Beijing, China; ^3^Department of Otolaryngology Head and Neck Surgery, Children's Hospital Capital Institute of Pediatrics, Beijing, China

**Keywords:** antibiotic exposure, asthma, adult, United States, NHANES

## Abstract

**Objectives:**

To investigate the relationship between antibiotic exposure and asthma in adults in the United States.

**Methods:**

Data was obtained from the National Health and Nutrition Examination Survey (NHANES) conducted between 1999 and 2018. A total of 51,124 participants were included, excluding those who were aged < 20 years, female participants who were pregnant, and individuals who did not complete the prescription medications questionnaire and the medical conditions questionnaire regarding asthma status. Antibiotic exposure was defined as the utilization of antibiotics within the past 30 days, categorized based on the Multum Lexicon Plus therapeutic classification system. Asthma was defined as having a history of asthma or having an asthma attack or wheezing symptoms in the past year.

**Results:**

The risk of asthma was found to be 2.557 (95% CI: 1.811, 3.612), 1.547 (95% CI: 1.190, 2.011) and 2.053 (95% CI: 1.344, 3.137) times greater in participants who had used macrolide derivatives, penicillin and quinolones in the past 30 days, respectively, compared with those not using antibiotics. After adjusting for demographic covariates and asthma-related factors, only macrolides derivatives were significantly associated with asthma in the 20–40 and 40–60 age groups. For individuals over 60 years old, quinolones were significantly associated with asthma. The effect of different types of antibiotic with asthma varied in male and female populations. Moreover, higher socioeconomic status, greater BMI, younger age, smoking habits, history of infection, chronic bronchitis, emphysema, and family history of asthma were all identified as risk factors for asthma.

**Conclusion:**

Our study indicated that three types of antibiotics were significantly associated with asthma in different subgroups of the population. Therefore, the use of antibiotics should be more strictly regulated.

## 1. Introduction

Asthma is a common chronic inflammatory disease of the airways. In 2019, it affected as many as 334 million people worldwide, and it continues to represent a major economic burden, in terms of both direct and indirect costs (1); In the United States, nearly 25.1 million people (7.8% of the population) had asthma in 2019, the associated mortality rate was 10.7 per million across people of all ages, and this rate was more than five times higher in adults (≥ 18 years) than children (< 18 years) (2). In addition, the prevalence of asthma has continued to increase during recent years. Therefore, it is important to understand the causes of asthma.

Previous studies have shown that maternal exposure to antibiotics during the prenatal period or lactation increases the risk of childhood asthma (3–6). In addition, in children, early life antibiotic exposure increases the risk of subsequent asthma. Babies who are administered various antibiotics within 6 months of birth are at higher risk of developing various allergic diseases in childhood, including food allergy, asthma, allergic dermatitis, allergic rhinitis, and conjunctivitis. Of these, asthma is the most closely associated with the use of antibiotics, followed by allergic rhinitis. The risk of allergic disease varies according to the antibiotic being administered; the highest risk was found to be associated with penicillin use (1.3 times higher) and the lowest risk with sulfonamide use (1.06 times higher) (7–11). Eight percent of children who were exposed to antibiotics between 0 and 11 months of age were found to have developed asthma by the age of 7 years, and the use of two or more antibiotics was associated with a 4.0% higher absolute risk of asthma. This relationship was found to be dose-dependent, and broad-spectrum antibiotic use was also found to be associated with a higher risk of asthma (12, 13). Finally, a population-based prospective cohort study conducted by Patrick et al. showed that a reduction in antibiotic use before 1 year of age is associated with a reduction in the incidence of asthma (14, 15). However, these previous studies focused on the relationship between antibiotic use and childhood asthma, and few have characterized the relationship between antibiotic use and asthma in adulthood.

Therefore, the aim of the study was to assess the relationship between antibiotic exposure and asthma in adults, using data obtained from NHANES 1999 to 2018.

## 2. Materials and methods

### 2.1. Participants

Data were obtained from the National Health and Nutrition Examination Survey (NHANES), a cross-sectional survey of a representative US population, which is led by the American National Centers for Health Statistics (NCHS) and the Centers for Disease Control and Prevention (CDC), and has been conducted every 2 years since 1999 (16). NHANES researchers use a complex multi-stage sampling design to obtain a representative, non-institutionalized sample of the population of the United States (17). The survey comprised face-to-face in-home interviews and physical examinations. Further details of the survey design and data files were available on NHANES website (16, 17).

**Figure 1:**
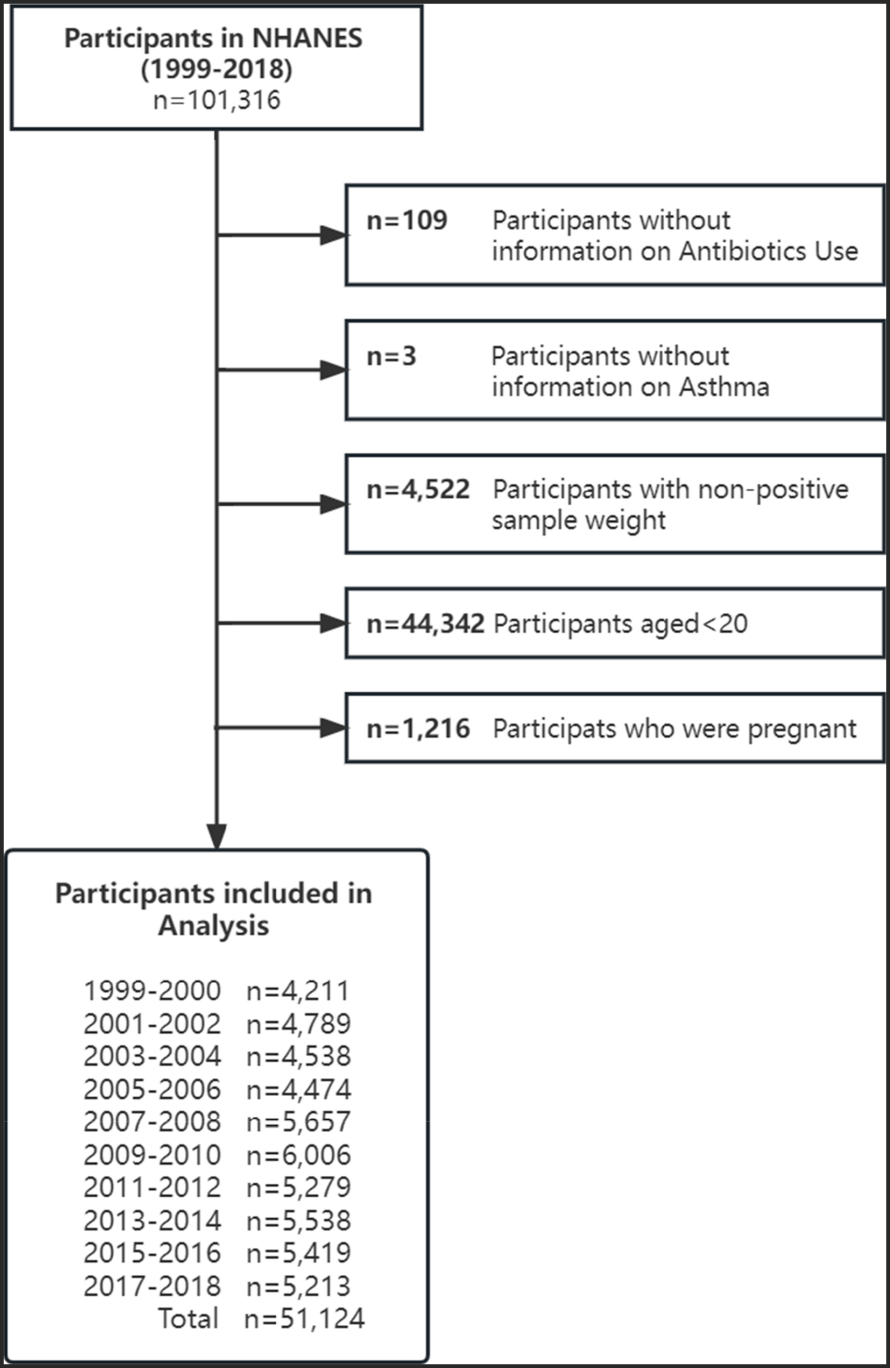
Flow diagram of included survey participants by survey year: National Health and Nutrition Examination Surveys (NHANES) from 1999 to 2018.

We included participants from 10 survey cycles of NHANES, between 1999 and 2018, and excluded those who were aged < 20 years (*n* = 44,342), did not complete the prescription medications questionnaire (*n* = 109) and the medical conditions questionnaire regarding asthma status (*n* = 3). In addition, female participants who were pregnant (*n* = 1,216) were also excluded. A total of 51,124 individuals were included in the study, with 25,170 (49.23%) being male and 25,954 (50.77%) being female. The detailed enrolment procedure is shown in Figure 1.

The protocols of NHANES were approved by NCHS Ethics Review Board, CDC (18), and written informed consent was obtained from all participants.

### 2.2. Definition of antibiotic use

During the in-home interviews, interviewers asked participants if they had taken any prescription medication during the preceding 30 days and exactly how they had administered it. If the participant replied in the affirmative, the interviewer asked them to provide the containers for the prescription medicines, and the names of the antibiotics, the durations of their use, and the reasons for each use were recorded. This information was reported by the participant when the containers were not available. The reported drug names were converted to standard generic names and encoded using the Multum Lexicon database (19). The drugs were placed into one of three categories according to the Multum Lexicon Plus therapeutic classification system, which is used to assign a therapeutic classification to each drug and its ingredients. In the present study, we focused on drugs with an antibiotic activity, which included those in the first-level category “anti-infective,” and the second-level category, which includes penicillin, cephalosporins, macrolide derivatives, quinolones, sulfonamides, tetracyclines, lincomycin derivatives, urological anti-infectives, and other antibiotics listed in the Multum Lexicon database. Because some of the drugs were less commonly used, the final four of the groups listed above were classified as “miscellaneous antibiotics” for the analysis. Non-antibiotics such as antiviral, antifungal, antituberculosis, anti-leprosy drugs, and topical antibiotics including those in ear and eye preparations, were excluded. Using penicillin, cephalosporins, macrolide derivatives, quinolones, sulfonamides, miscellaneous antibiotics during the preceding 30 days and those did not use antibiotics were regarded as multi-classified variable.

### 2.3. Assessment of asthma

The information regarding asthma and related symptoms collected by NHANES through a self-administered questionnaires, which were completed either during the in-home interview or clinic visit. Asthma was defined by respondents giving a positive response to either of three following questions: “Has a doctor or other health professional ever told you that you have asthma?,” “In the past 12 months have you had wheezing or whistling in your chest?” and “During the past 12 months, have you had an episode of asthma or an asthma attack?” (20).

### 2.4. Covariates

Data regarding covariates were obtained through interviews conducted at home and physical examinations performed at the MECs. Age, sex, ethnicity, household socioeconomic status, educational level, health insurance coverage status, smoking status, alcohol consumption status, history of diseases (including infection, chronic bronchitis and emphysema), and family history of asthma were self-reported by the participants during the interview. Participants were categorized according to age as adult (age 20–40 years), middle-aged (aged 40–60 years), and older (aged > 60 years). Ethnicity was categorized as Non-Hispanic white people, Non-Hispanic black people, Hispanic people, and other. Household socioeconomic status was assessed using the family income-to-poverty ratio (family PIR), which was calculated by dividing household income by the poverty threshold for the survey year (21). This result varied according to family size and geographic location (22). The PIR was used to define three categories of socioeconomic status: low (PIR ≤ 1.3), medium (1.3 < PIR ≤ 3.5), and high (PIR > 3.5). Educational level was classified as pre-high school, high school graduate or general educational development (GED), and college or above. A participant was defined as “Covered by Health Insurance” if they had health insurance or another type of healthcare plan, such as health insurance obtained through their employment or purchased directly, or government programs, such as Medicare and Medicaid, which provide medical care or help with medical bills. The participants were also categorized as being current smokers, former smokers, or never having been a smoker of cigarettes. The alcohol consumption of a participant was judged by their response to the question “Do you have at least 12 alcoholic beverages per year?.” A standard drink was considered to be 12 fl. oz. of beer, 5 fl. oz. of wine, or 1.5 fl. oz. of liquor (23), and a response in the affirmative led to the participant being recorded as “Having a history of alcohol consumption.” A history of infection was identified using the participant’s self-reported current health status, and individuals were recorded as “having a history of infection” if they had experienced influenza, pneumonia, or ear infection that started during the preceding 30 days. Body mass index (BMI) was calculated as body mass (kg)/height (m) squared. Height and body mass were measured at the MECs according to a standard operating procedure. Individuals with BMI ≥ 25 kg/m^2^ were recorded as being “Overweight or Obese” and those with BMI ≥ 18.5 kg/m^2^ and < 25 kg/m^2^ were recorded as being of “Normal weight.”

### 2.5. Statistical analysis

Statistical analyses were performed using the 20-year sampling weights associated with the MECs, according to the NCHS Analytic Guidelines, unless otherwise specified (24). The sampling weights associated with the MECs were calculated using the inequality of the sampling probabilities for selection in the present study and the non-response rates of participants in the NHANES as a whole and at the MECs specifically. Geometric means and 95% confidence intervals (CIs) are used to summarize continuous data, and numbers and percentages are used to summarize categorical data. The demographic and physical characteristics of the asthma and non-asthma groups were compared using the Rao-Scott *χ*^2^ test.

Weighted logistic regression models were used to assess the relationship between asthma and the use of prescription antibiotics. Three models were created: model 1 was not adjusted; model 2 adjusted for age, sex, ethnicity, educational level, socioeconomic status, health insurance status, and BMI; and model 3 further adjusted for smoking status, history of diseases (including infection, bronchitis and emphysema), and family history of asthma. Odds ratios (ORs) and their 95% CIs were used to quantify the relationship between asthma and the type of antibiotic being used. In addition, subgroup analyses according to sex, ethnicity, and age were performed. When creating the model stratified according to sex, all the covariates adjusted for in model 3 were used, with the exception of sex. Analogous processes were used for the subgroup analyses according to age and ethnicity.

All the analyses were two-sided, and a *p* value of < 0.05 was considered to represent statistical significance. SAS v.9.4 (SAS Institute, Cary, NC, United States) and R v.4.1.3 (R Project for Statistical Computing, Vienna, Austria) were used for the statistical analyses.

## 3. Results

### 3.1. Baseline characteristics of the participants

As demonstrated in Table 1, the prevalence of asthma was 18.83% (9,627/51,124) and the overall prevalence of antibiotic use was 3.81% (1,946/51,124). The weighted mean age of the participants with asthma was 46.2 years, which was significantly lower than the weighted mean age of those without asthma (47.5 years, *p* < 0.001). Out of the 51,124 participants, 16,788 (32.84%) were aged between 20 and 40 years, 16,542 (32.36%) were aged between 40 and 60 years, and 17,794 (34.81%) were aged > 60 years. Non-Hispanic white people were the most common ethnicity (approximately 43.93%) while “other ethnicities,” including Asian, was the least common. Significant differences were found between the asthma and non-asthma groups with respect to most of the parameters assessed. Among the six categories of antibiotics included in the analysis, penicillin had the highest weighted prevalence of use (1.13%) and sulfonamide antibiotics had the lowest (0.31%).

**Table 1:** Characteristics of participants aged over 20 by asthma status: NHANES 1999–2018.

Variables	Non-asthma group	Asthma group	Rao-Scott *χ*^2^	*p* Value^b^
Sample size	*n* = 41,497 (81.17)	*n* = 9,627 (18.83)		
Age, year	47.50 ± 18.17	46.21 ± 17.95	3.99	<0.0001
Used antibiotics in the past 30 days			62.16	<0.0001
Cephalosporins	174 (0.42)	66 (0.69)		
Macrolides derivatives	134 (0.32)	93 (0.97)		
Penicillin	426 (1.03)	153 (1.59)		
Quinolones	153 (0.37)	71 (0.74)		
Sulfonamides	119 (0.29)	40 (0.42)		
Miscellaneous antibiotics	386 (0.93)	131 (1.36)		
Not used antibiotics	40,105 (96.65)	9,073 (94.25)		
Age, year^a^			14.71	<0.0001
20–40	13,497 (32.53)	3,291 (34.19)		
40–60	13,364 (32.20)	3,178 (33.01)		
≥ 60	14,636 (35.27)	3,158 (32.80)		
Sex			52.42	<0.0001
Male	20,800 (50.12)	4,370 (45.39)		
Female	20,697 (49.88)	5,257 (54.61)		
Ethnicity			117.27	<0.0001
Non-hispanic white	17,617 (42.45)	4,843 (50.31)		
Non-hispanic black	8,626 (20.79)	2,251 (23.38)		
Hispanic	11,207 (27.01)	1836 (19.07)		
Other	4,047 (9.75)	697 (7.24)		
Family income to poverty ratio (PIR)			105.56	<0.0001
≤1.30	10,928 (29.29)	3,164 (36.10)		
1.30–3.50	14,555 (39.01)	3,180 (36.28)		
>3.50	11,832 (31.71)	2,420 (27.61)		
Education			63.08	<0.0001
Less than high school	20,974 (50.62)	4,865 (50.58)		
High school graduates or general education development (GED)	11,358 (27.41)	2,998 (31.17)		
Some college or above	9,105 (21.97)	1755 (18.25)		
Body mass index			28.09	<0.0001
Underweight	1,497 (3.61)	373 (3.87)		
Normal weight	11,840 (28.53)	2,300 (23.89)		
Overweight or obesity	28,160 (67.86)	6,954 (72.23)		
Smoking status			484.30	<0.0001
Non-smoker	20,828 (56.25)	3,791 (42.90)		
Former smoker	9,078 (24.52)	2,318 (26.23)		
Current smoker	7,124 (19.24)	2,728 (30.87)		
Covered by health Insurance			16.81	<0.0001
No	8,785 (21.26)	1857 (19.37)		
Yes	32,533 (78.74)	7,729 (80.63)		
Had flu, pneumonia, ear infection during past 30 days?			190.61	<0.0001
No	36,139 (96.24)	8,214 (92.83)		
Yes	1,410 (3.76)	634 (7.17)		
Had chronic bronchitis			1911.85	<0.0001
No	40,316 (97.15)	7,795 (80.97)		
Yes	1,181 (2.85)	1832 (19.03)		
Had emphysema			772.93	<0.0001
No	41,087 (99.01)	8,974 (93.22)		
Yes	410 (0.99)	653 (6.78)		
Family history of asthma			1302.10	<0.0001
No	34,704 (83.63)	6,100 (63.36)		
Yes	6,793 (16.37)	3,527 (36.64)		

a Age group 20–40 included participants aged ≥ 20 and < 40; 40–60 included participants aged ≥ 40 and < 60.

b *p* Values were based on Rao-Scott Chi-square tests for categorized variables and weighted Student-*t* tests for continuous variables to test the difference of frequencies across asthma status groups.

Values were survey-weighted frequencies, and percentage in colons (%) in parenthesis.

### 3.2. Relationship between asthma and antibiotic use

The model 1 indicates that, compared to those not using antibiotics, individuals who used macrolide derivatives, penicillin, or quinolones had a 2.557 (95% CI: 1.811, 3.612), 1.547 (95% CI: 1.190, 2.011) and 2.053 (95% CI: 1.344, 3.137) times greater risk of asthma, respectively. However, after adjusting for demographic covariates and asthma-related factors, but only macrolides derivatives remained significantly associated with asthma (Table 2, model 3). No significant association was found between the use of the other three types of antibiotics and asthma, either before or after controlling for covariates (Table 2). The results also revealed that female participants had a 14.5% higher risk of asthma than male participants (OR: 1.145, 95% CI: 1.072, 1.223). Recent infection (OR:1.891, 95%CI: 1.648, 2.169), chronic bronchitis (OR:5.622, 95%CI: 4.954, 6.380) and emphysema (OR:4.102, 3.273, 5.141) were found to increase the risk of asthma. In addition, Hispanic participants, non-Hispanic black people and other ethnicities were all at a lower risk of asthma than non-Hispanic white people. Moreover, smoking was found to be a risk factor for asthma, with former and current smokers were at 24.3 and 78.6%, respectively, higher risks of asthma than never-smokers (former smoker, OR: 1.243, 95% CI: 1.146, 1.348; current smoker, OR: 1.786, 95% CI: 1.626, 1.961). Higher socioeconomic status, greater BMI, younger age and family history of asthma were all found to be risk factors for asthma (Supplementary Table S1).

**Table 2:** Association of asthma and antibiotics use among American adults: NHANES 1999–2018.

	Model 1				Model 2				Model 3			
			95%CI				95%CI				95%CI	
	OR	*p*	Lower	Upper	OR	*p*	Lower	Upper	OR	*p*	Lower	Upper
Not used antibiotics	ref	/	/	/	ref	/	/	/	ref	/	/	/
Macrolides derivatives	2.557	0.001	1.811	3.612	2.593	<0.001	1.831	3.671	1.983	0.008	1.341	2.930
Sulfonamides	1.112	0.180	0.690	1.790	1.032	0.184	0.607	1.755	0.862	0.219	0.482	1.544
Penicillin	1.547	0.691	1.190	2.011	1.423	0.919	1.085	1.864	1.235	0.738	0.934	1.634
Cephalosporins	1.339	0.553	0.937	1.915	1.257	0.506	0.863	1.830	1.092	0.679	0.717	1.663
Miscellaneous antibiotics	1.230	0.169	0.926	1.632	1.099	0.080	0.807	1.498	0.986	0.282	0.683	1.423
Quinolones	2.053	0.077	1.344	3.137	2.038	0.051	1.317	3.155	1.369	0.458	0.844	2.220

Model 1 was not adjusted; Model 2 adjusted for age, sex, ethnicity, educational level, socioeconomic status, health insurance status, and BMI; Model 3 further adjusted for smoking status, history of diseases (including infection, bronchitis and emphysema), and family history of asthma.

OR: odds ratio. CI: confidence interval.

### 3.3. Results of the subgroup analysis of the relationship between asthma and antibiotic use according to sex, age, and ethnicity

**Figure 2:**
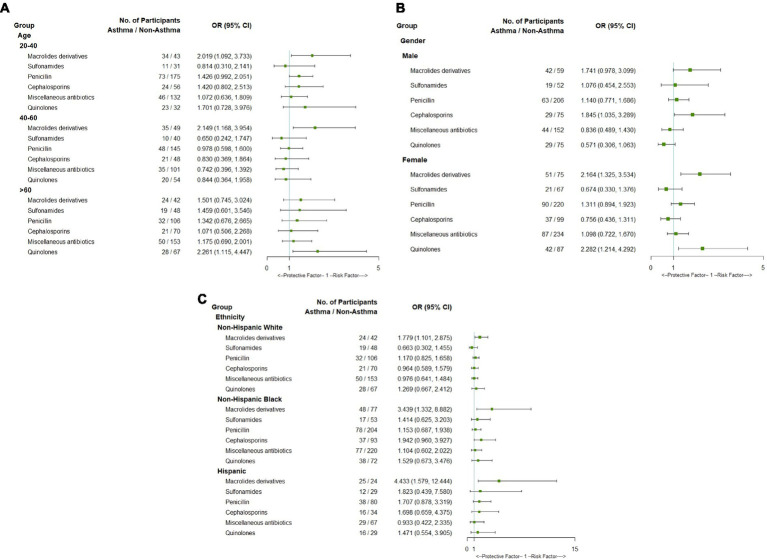
Forest plot of subgroup analysis of association between asthma and antibiotics use by age **(A)**, gender **(B)** and ethnicity **(C)**.

Subgroup analyses were conducted based on demographic characteristics including sex, age, and ethnicity (Figure 2). The macrolide derivatives were closely associated with asthma in adults aged between 20 and 40 and between 40 and 60, but not among those aged > 60. Conversely, the use of quinolones was significantly associated with an increased risk of asthma in individuals aged over 60 years (Figure 2A). In addition, the use of macrolide derivatives or quinolones was significantly associated with asthma in women, while only cephalosporins had significant association with asthma in men (Figure 2B). There was a significant association between macrolide derivatives and asthma in all three races, while other antibiotics did not (Figure 2C). Interaction analyses between sex and age were also performed, and it was found that quinolone was associated with a lower risk of asthma in men between the ages of 20 and 40, but not in men of other age groups. Additionally, the association between quinolones and asthma was statistically significant in women aged between 20 and 40 and over 60, while the use of macrolides was associated with a high correlation of asthma in middle-aged women aged 40–60 (Supplementary Table S2).

## 4. Discussion

Asthma has been recognized for more than 1,800 years but has become of particular concern during the past four decades, and our understanding of the underlying pathophysiology and the various clinical presentations has developed rapidly. However, it remains one of the most important non-communicable diseases, and causes substantial disability and death worldwide. Therefore, it requires global attention and commitment to lessen its impact. Excessive sensitivity of the airway to external stimuli (hyper-responsiveness) and airway inflammation have been considered to lead to asthma. However, in recent years, the relationship between the microbiota and asthma has become of great interest, and antibiotics have clear effects on the gut and lung microbiota (14, 25–27). Moreover, animal experiments have shown that most antibiotics have adverse effects on allergic diseases, increase susceptibility to allergic respiratory diseases, and aggravate asthma (28–30).

Previous studies have shown that antibiotic use during pregnancy and within the first year after birth increase the risk of asthma in children, but less research has focused on the relationship between antibiotic use and asthma in adults. The present findings provide evidence for an association between antibiotic exposure and asthma in adults. Moreover, we found that macrolide derivatives specifically were associated with asthma, even after adjustment for covariates. Azithromycin, a macrolide antibiotic, is often prescribed to ameliorate asthma and improve the quality of life of patients with asthma (31–34). However, its use has been associated with a significant increase in macrolide resistance genes, and some macrolides have severe side effects, such as a higher risk of cardiovascular events (35, 36). Therefore, further causality research is needed to better understand the relationship between macrolides and asthma.

In the present study, we found that the risk of asthma was significantly higher in individuals who had recent infection during the preceding 30 days, chronic bronchitis and emphysema than in those who had not. This is consistent with the results of Sandrock et al. (37), who found that infectious agents are the principal triggers of chronic persistent asthma and severe acute exacerbations of bronchial asthma. Respiratory infections caused by agents such as viruses, *Chlamydophila*, or *Mycoplasma* are associated with wheezing illnesses in individuals of all ages and may affect the incidence and severity of asthma, and such infections have been hypothesized to have significant roles in the pathogenesis of asthma (38). Therefore, prevention of infections, chronic bronchitis and emphysema is an effective way to control asthma.

Consistent with the findings of Pietinalho et al. (39) that both passive exposure to environmental tobacco smoke and active smoking can worsen asthma in children and adults, we found that smoking, whether past or present, is a risk factor for asthma in adults; The whole society should participate in the control of smoking. Previous studies have demonstrated that obesity is both a major risk factor for asthma and a disease modifier of asthma, regardless of age; and we also found that higher educational level, socioeconomic status, greater BMI, younger age and family history of asthma are closely related to asthma. Furthermore, individuals of differing ethnicity show different relationships between antibiotic use and asthma. Macrolide use was found to have a closely associated with asthma in all three race. These covariates appeared to affect the relationship between antibiotic use and asthma in the sample, which may suggest that antibiotics contribute to the risk of asthma through differing biological mechanisms.

In summary, our findings demonstrate an association between antibiotic exposure and asthma in adults in the United States. The government should play an important role in solving the problem of asthma, including in encouraging a healthy lifestyle and reducing tobacco consumption; identifying and avoiding the predisposing factors of asthma is essential to prevent and minimize asthma attacks. In addition, multidisciplinary studies are needed to increase understanding of asthma, including the causes, triggers, and effective management measures. However, the present study had several limitations. First, because it was a cross-sectional study, inferences regarding causal links between antibiotic exposure and asthma cannot be made. In addition, we studied the association with the use of antibiotics within the preceding 30 days, and only small numbers of participants were taking some of the categories of antibiotics. Second, information on antibiotic use, such as the type, dose, route of administration, and indication, was not available. This is of significance because high cumulative doses, administration during early pregnancy, and the use of a broad-spectrum antibiotic have been shown to be associated with childhood asthma risk, but information regarding adults is lacking (40). Third, we did not consider the contribution of antibiotic residues in food, especially meat, and water, which may have been present because subclinical doses of antibiotics are used to promote animal growth (41–44). Fourth, the association between asthma and antibiotic exposure is just one component of a complex network. In addition to diet, environment, genetics, upper respiratory tract infection, some medicines, exercise, and emotional stress are etiological factors for asthma (45–47). Therefore, more comprehensive studies are necessary to verify the relationship between antibiotic use and asthma. Fifth, the data for asthma and antibiotic use were self-reported, and therefore may not have been highly accurate.

In conclusion, we investigated the relationship between antibiotic exposure and asthma in adult populations in the United States, and found that certain types of antibiotics were associated with a higher risk of asthma in specific subgroups of the population. These findings imply that we should more strictly regulate the use of antibiotics. More research should be done in the future to find out how the microbiota is distributed in adults with asthma who used antibiotics, like macrolides, and how macrolides affect asthma mechanically.

## Data availability statement

Publicly available datasets were analyzed in this study. This data can be found at: https://www.cdc.gov/nchs/nhanes/index.htm.

## Ethics statement

The studies involving human participants were reviewed and approved by NCHS Ethics Review Board. The patients/participants provided their written informed consent to participate in this study.

## Author contributions

JL and TX designed the study and led the writing of the paper. SL and FC drafted the manuscript. FC and GH did the data analysis. YC, TL, and DH accessed and verified the data. CH and WL critically revised the manuscript. All authors contributed to the article and approved the submitted version.

## Funding

This study was supported by the National Natural Science Foundation (32070188) and Public service development and reform pilot project of Beijing Medical Research Institute (BMR2021-3); Research Foundation of Capital Institute of Pediatrics (ERB-2023-01). The funders had no role in the study design, data collection and interpretation or the decision to submit the work for publication.

## Conflict of interest

The authors declare that the research was conducted in the absence of any commercial or financial relationships that could be construed as a potential conflict of interest.

## Publisher’s note

All claims expressed in this article are solely those of the authors and do not necessarily represent those of their affiliated organizations, or those of the publisher, the editors and the reviewers. Any product that may be evaluated in this article, or claim that may be made by its manufacturer, is not guaranteed or endorsed by the publisher.

## References

[1] Enilari O Sinha S. The global impact of asthma in adult populations. Ann Glob Health. (2019) 85:2. doi: 10.5334/aogh.2412, PMID: 30741503 PMC7052341

[2] CDC. Asthma: Most Recent National Asthma Data. Atlanta, GA: US Department of Health and Human Services, CDC (2021).

[3] Souza da Cunha S Santorelli G Pearce N Wright J Oddie S Petherick E. Evidence for causal associations between prenatal and postnatal antibiotic exposure and asthma in children, England. Clin Exp Allergy. (2021) 51:1438–48. doi: 10.1111/cea.13999, PMID: 34363720

[4] Loewen K Monchka B Mahmud SM TJong G Azad MB. Prenatal antibiotic exposure and childhood asthma: a population-based study. Eur Respir J. (2018) 52:1702070. doi: 10.1183/13993003.02070-2017, PMID: 29678946

[5] Renz H Skevaki C. Early life microbial exposures and allergy risks: opportunities for prevention. Nat Rev Immunol. (2021) 21:177–91. doi: 10.1038/s41577-020-00420-y, PMID: 32918062

[6] Simioni J Hutton EK Gunn E Holloway AC Stearns JC McDonald H. A comparison of intestinal microbiota in a population of low-risk infants exposed and not exposed to intrapartum antibiotics: the Baby & Microbiota of the intestine cohort study protocol. BMC Pediatr. (2016) 16:183. doi: 10.1186/s12887-016-0724-5, PMID: 27832763 PMC5103394

[7] Zven SE Susi A Mitre E Nylund CM. Association between use of multiple classes of antibiotic in infancy and allergic disease in childhood. JAMA Pediatr. (2020) 174:199–200. doi: 10.1001/jamapediatrics.2019.4794, PMID: 31860016 PMC6990912

[8] Mitre E Susi A Kropp LE Schwartz DJ Gorman GH Nylund CM. Association between use of acid-suppressive medications and antibiotics during infancy and allergic diseases in early childhood. JAMA Pediatr. (2018) 172:e180315. doi: 10.1001/jamapediatrics.2018.0315, PMID: 29610864 PMC6137535

[9] Johnson CC Ownby DR. The infant gut bacterial microbiota and risk of pediatric asthma and allergic diseases. Transl Res. (2017) 179:60–70. doi: 10.1016/j.trsl.2016.06.010, PMID: 27469270 PMC5555614

[10] Carstens LE Westerbeek EA van Zwol A van Elburg RM. Neonatal antibiotics in preterm infants and allergic disorders later in life. Pediatr Allergy Immunol. (2016) 27:759–64. doi: 10.1111/pai.12614, PMID: 27434167

[11] Aversa Z Atkinson EJ Schafer MJ Theiler RN Rocca WA Blaser MJ. Association of infant antibiotic exposure with childhood health outcomes. Mayo Clin Proc. (2021) 96:66–77. doi: 10.1016/j.mayocp.2020.07.019, PMID: 33208243 PMC7796951

[12] Toivonen L Schuez-Havupalo L Karppinen S Waris M Hoffman KL Camargo CA. Antibiotic treatments during infancy, changes in nasal microbiota, and asthma development: population-based cohort study. Clin Infect Dis. (2021) 72:1546–54. doi: 10.1093/cid/ciaa262, PMID: 32170305 PMC8096219

[13] Zhang X Borbet TC Fallegger A Wipperman MF Blaser MJ Müller A. An antibiotic-impacted microbiota compromises the development of colonic regulatory T cells and predisposes to dysregulated immune responses. MBio. (2021) 12:e03335–20. doi: 10.1128/mBio.03335-20, PMID: 33531385 PMC7858066

[14] Patrick DM Sbihi H Dai DLY Al Mamun A Rasali D Rose C. Decreasing antibiotic use, the gut microbiota, and asthma incidence in children: evidence from population-based and prospective cohort studies. Lancet Respir Med. (2020) 8:1094–105. doi: 10.1016/S2213-2600(20)30052-7, PMID: 32220282

[15] McDonnell L Gilkes A Ashworth M Rowland V Harries TH Armstrong D. Association between antibiotics and gut microbiome dysbiosis in children: systematic review and meta-analysis. Gut Microbes. (2021) 13:1–18. doi: 10.1080/19490976.2020.1870402, PMID: 33651651 PMC7928022

[16] Centers for Disease Control and Prevention NCHS. NHANES 1999–2000 overview (2002). Available at: https://wwwn.cdc.gov/nchs/nhanes/continuousnhanes/releasenotes.aspx?BeginYear=1999. Accessed November 15, 2022.

[17] Zipf G Chiappa M Porter KS Ostchega Y Lewis BG Dostal J. National health and nutrition examination survey: plan and operations, 1999-2010. Vital Health Stat Ser 1, Programs Collect Proced. (2013) 56:1–37. 25078429

[18] Centers for Disease Control and Prevention. NCHS ethics review board (ERB) approval (2021). Available at: https://www.cdc.gov/nchs/nhanes/irba98.htm

[19] database ML. National Health and nutrition examination survey: 1999–2018 data documentation, codebook, and frequencies (2021). Available at: https://www.cerner.com/solutions/drug-database. Accessed November 15, 2022.

[20] Lødrup Carlsen KC Roll S Carlsen KH Mowinckel P Wijga AH Brunekreef B. Does pet ownership in infancy lead to asthma or allergy at school age? Pooled analysis of individual participant data from 11 European birth cohorts. PLoS One. (2012) 7:e43214. doi: 10.1371/journal.pone.0043214, PMID: 22952649 PMC3430634

[21] Fadeyev K Nagao-Sato S Reicks M. Nutrient and food group intakes among U.S. children (2-5 years) differ by family income to poverty ratio, NHANES 2011-2018. Int J Environ Res Public Health. (2021) 18:11938. doi: 10.3390/ijerph182211938, PMID: 34831692 PMC8622378

[22] Services USDoHH. Poverty Guidelines, Research, and Measurement. Washington, DC: U.S. Department of Health & Human Services (2022).

[23] Centers for Disease Control and Prevention NCfHS. National Health and nutrition examination survey-alcohol use (2022). http://wwwn.cdc.gov/Nchs/Nhanes/2009-2010/ALQ_F.htm. Accessed November 28, 2022.

[24] Ingram DD Malec DJ Makuc DM Kruszon-Moran D Gindi RM Albert M. National Center for Health Statistics guidelines for analysis of trends. Vital Health Stat Ser 2, Data Eval Meth Res. (2018) 179:1–71. 29775435

[25] Hufnagl K Pali-Schöll I Roth-Walter F Jensen-Jarolim E. Dysbiosis of the gut and lung microbiome has a role in asthma. Semin Immunopathol. (2020) 42:75–93. doi: 10.1007/s00281-019-00775-y, PMID: 32072252 PMC7066092

[26] Liu C Makrinioti H Saglani S Bowman M Lin LL Camargo CA Jr. Microbial dysbiosis and childhood asthma development: integrated role of the airway and gut microbiome, environmental exposures, and host metabolic and immune response. Front Immunol. (2022) 13:1028209. doi: 10.3389/fimmu.2022.1028209, PMID: 36248891 PMC9561420

[27] Stokholm J. Can perturbations in microbial maturation cause asthma? Lancet Respir Med. (2020) 8:1063–5. doi: 10.1016/S2213-2600(20)30002-3, PMID: 32220281

[28] Wypych TP Marsland BJ. Antibiotics as instigators of microbial Dysbiosis: implications for asthma and allergy. Trends Immunol. (2018) 39:697–711. doi: 10.1016/j.it.2018.02.008, PMID: 29655522

[29] Skalski JH Limon JJ Sharma P Gargus MD Nguyen C Tang J. Expansion of commensal fungus *Wallemia mellicola* in the gastrointestinal mycobiota enhances the severity of allergic airway disease in mice. PLoS Pathog. (2018) 14:e1007260. doi: 10.1371/journal.ppat.1007260, PMID: 30235351 PMC6147580

[30] Yang X Feng H Zhan X Zhang C Cui R Zhong L. Early-life vancomycin treatment promotes airway inflammation and impairs microbiome homeostasis. Aging (Albany NY). (2019) 11:2071–81. doi: 10.18632/aging.101901, PMID: 30981206 PMC6503881

[31] Gibson PG Yang IA Upham JW Reynolds PN Hodge S James AL. Effect of azithromycin on asthma exacerbations and quality of life in adults with persistent uncontrolled asthma (AMAZES): a randomised, double-blind, placebo-controlled trial. Lancet. (2017) 390:659–68. doi: 10.1016/S0140-6736(17)31281-3, PMID: 28687413

[32] Brusselle G Pavord I. Azithromycin in uncontrolled asthma. Lancet. (2017) 390:629–30. doi: 10.1016/S0140-6736(17)31547-7, PMID: 28687412

[33] Stanbrook MB. Azithromycin reduced exacerbations and improved QoL in symptomatic asthma despite inhaled maintenance therapy. Ann Intern Med. (2017) 167:JC42. doi: 10.7326/ACPJC-2017-167-8-042, PMID: 29049761

[34] Crooks MG Faruqi S Morice AH. How does azithromycin improve asthma exacerbations? Lancet. (2018) 391:28. doi: 10.1016/S0140-6736(17)33318-4 29323653

[35] Taylor SL Leong LEX Mobegi FM Choo JM Wesselingh S Yang IA. Long-term azithromycin reduces *Haemophilus influenzae* and increases antibiotic resistance in severe asthma. Am J Respir Crit Care Med. (2019) 200:309–17. doi: 10.1164/rccm.201809-1739OC, PMID: 30875247

[36] Li H Liu DH Chen LL Zhao Q Yu YZ Ding JJ. Meta-analysis of the adverse effects of long-term azithromycin use in patients with chronic lung diseases. Antimicrob Agents Chemother. (2014) 58:511–7. doi: 10.1128/AAC.02067-13, PMID: 24189261 PMC3910718

[37] Sandrock CE Norris A. Infection in severe asthma exacerbations and critical asthma syndrome. Clin Rev Allergy Immunol. (2015) 48:104–13. doi: 10.1007/s12016-014-8435-x 24984968

[38] Guilbert TW Denlinger LC. Role of infection in the development and exacerbation of asthma. Expert Rev Respir Med. (2010) 4:71–83. doi: 10.1586/ers.09.60, PMID: 20305826 PMC2840256

[39] Pietinalho A Pelkonen A Rytilä P. Linkage between smoking and asthma. Allergy. (2009) 64:1722–7. doi: 10.1111/j.1398-9995.2009.02208.x 19832738

[40] Turi KN Gebretsadik T Ding T Abreo A Stone C Hartert TV. Dose, timing, and Spectrum of prenatal antibiotic exposure and risk of childhood asthma. Clin Infect Dis. (2021) 72:455–62. doi: 10.1093/cid/ciaa085, PMID: 31994697 PMC7850553

[41] Van Boeckel TP Brower C Gilbert M Grenfell BT Levin SA Robinson TP. Global trends in antimicrobial use in food animals. Proc Natl Acad Sci U S A. (2015) 112:5649–54. doi: 10.1073/pnas.1503141112, PMID: 25792457 PMC4426470

[42] Van Boeckel TP Pires J Silvester R Zhao C Song J Criscuolo NG. Global trends in antimicrobial resistance in animals in low-and middle-income countries. Science. (2019) 365. doi: 10.1126/science.aaw1944 31604207

[43] Zhang Y Tang W Wang Y Nian M Jiang F Zhang J. Environmental antibiotics exposure in school-age children in Shanghai and health risk assessment: a population-based representative investigation. Sci Total Environ. (2022) 824:153859. doi: 10.1016/j.scitotenv.2022.153859, PMID: 35176387

[44] Wang C Zhao Y Liu S Xiao Q Liang W Song Y. Contamination, distribution, and risk assessment of antibiotics in the urban surface water of the Pearl River in Guangzhou, South China. Environ Monit Assess. (2021) 193:98. doi: 10.1007/s10661-021-08887-5, PMID: 33511434

[45] Lemanske RF Jr Busse WW. Asthma. JAMA. (1997) 278:1855–73. 45. 9396647

[46] Barnthouse M Jones BL. The impact of environmental chronic and toxic stress on asthma. Clin Rev Allergy Immunol. (2019) 57:427–38. doi: 10.1007/s12016-019-08736-x 31079340

[47] Ntontsi P Photiades A Zervas E Xanthou G Samitas K. Genetics and epigenetics in asthma. Int J Mol Sci. (2021) 22:2412. doi: 10.3390/ijms22052412, PMID: 33673725 PMC7957649

