# Supplementary material for: Relationships between antibiotic exposure and asthma in adults in the United States: results of the National Health and Nutrition Examination Survey between 1999 and 2018
Source: Front Public Health. 2023 Apr 27;11:1123555. doi: 10.3389/fpubh.2023.1123555 (PMC10173742; doi:10.3389/fpubh.2023.1123555)
Supplement: Supplementary file 1 [file Data_Sheet_1.pdf]

Supplementary Table S1. Association of asthma and Antibiotics use among American adults: NHANES 1999-2018

Variables	*β*	*t*	*p* Value	OR	Lower 95%CI	Upper 95%CI
**Model 1**						
Antibiotics (Not Use Antibiotics)	Ref	/	/	/	/	/
Antibiotics (Macrolides derivatives)	0.553	3.29	0.001	2.557	1.811	3.612
Antibiotics (Sulfonamides)	-0.280	-1.35	0.180	1.112	0.690	1.790
Antibiotics (Penicillin)	0.050	0.40	0.691	1.547	1.190	2.011
Antibiotics (Cephalosporins)	-0.093	-0.59	0.552	1.339	0.937	1.915
Antibiotics (Miscellaneous antibiotics)	-0.179	-1.38	0.169	1.230	0.926	1.632
Antibiotics (Quinolones)	0.334	1.78	0.077	2.053	1.344	3.137
**Model 2**						
Antibiotics (Macrolides derivatives)	0.614	3.59	<.001	2.593	1.831	3.671
Antibiotics (Sulfonamides)	-0.308	-1.33	0.184	1.032	0.607	1.755
Antibiotics (Penicillin)	0.014	0.10	0.919	1.423	1.085	1.864
Antibiotics (Cephalosporins)	-0.110	-0.67	0.506	1.257	0.863	1.830
Antibiotics (Miscellaneous antibiotics)	-0.244	-1.76	0.080	1.099	0.807	1.498
Antibiotics (Quinolones)	0.373	1.97	0.051	2.038	1.317	3.155
Ethnicity (Hispanic)	-0.346	-8.56	<.001	0.556	0.503	0.613
Ethnicity (Non-Hispanic Black)	0.101	3.33	0.001	0.868	0.807	0.934
Ethnicity (Other)	0.003	0.06	0.951	0.788	0.690	0.899
Sex (Female)	0.100	6.53	<.001	1.222	1.150	1.299
Covered by health insurance	0.042	1.78	0.076	1.088	0.991	1.194
Age (40-60)	0.032	1.29	0.199	0.923	0.856	0.996
Age (>60)	-0.144	-5.28	<.001	0.774	0.712	0.842
BMI (Overweight or Obesity)	0.095	3.22	0.002	1.229	1.145	1.318
BMI (Underweight)	0.016	0.29	0.773	1.134	0.959	1.342
Family PIR (1.30-3.50)	-0.066	-3.09	0.002	0.720	0.671	0.773
Family PIR (>3.50)	-0.196	-7.66	<.001	0.633	0.581	0.689
Education (College or above)	-0.151	-5.02	<.001	0.810	0.736	0.892
Education (High School Graduates or GED)	0.091	3.87	<.001	1.032	0.961	1.109
**Model 3**						
Antibiotics (Macrolides derivatives)	0.522	2.71	0.008	1.983	1.341	2.930
Antibiotics (Sulfonamides)	-0.310	-1.23	0.219	0.862	0.482	1.544
Antibiotics (Penicillin)	0.049	0.33	0.738	1.235	0.934	1.634
Antibiotics (Cephalosporins)	-0.074	-0.42	0.679	1.092	0.717	1.663
Antibiotics (Miscellaneous antibiotics)	-0.176	-1.08	0.282	0.986	0.683	1.423
Antibiotics (Quinolones)	0.152	0.74	0.458	1.369	0.844	2.220
Ethnicity (Hispanic)	-0.246	-6.24	<.001	0.666	0.602	0.737
Ethnicity (Non-Hispanic Black)	0.074	2.41	0.017	0.917	0.848	0.991
Ethnicity (Other)	0.012	0.23	0.818	0.862	0.755	0.983
Sex (Female)	0.068	4.06	<.001	1.145	1.072	1.223
Covered By Health Insurance (Yes)	0.055	2.09	0.038	1.116	1.006	1.239

Age (40-60)	-0.002	-0.08	0.937	0.842	0.771	0.921
Age (>60)	-0.167	-5.27	<.001	0.715	0.647	0.789
BMI (Overweight or Obesity)	0.138	3.62	<.001	1.253	1.163	1.350
BMI (Underweight)	-0.050	-0.73	0.465	1.039	0.847	1.276
Family PIR (1.30-3.50)	-0.070	-3.00	0.003	0.810	0.743	0.882
Family PIR (>3.50)	-0.071	-2.39	0.018	0.809	0.727	0.900
Smoke (Current smoker)	0.314	10.14	<.001	1.786	1.626	1.961
Smoke (Former smoker)	-0.048	-1.74	0.083	1.243	1.146	1.348
Infection (Yes)	0.319	9.17	<.001	1.891	1.648	2.169
Education (College or above)	-0.037	-1.14	0.255	0.973	0.879	1.077
Education (High School Graduates or GED)	0.046	1.72	0.087	1.057	0.975	1.145
Bronchitis (Yes)	0.863	26.96	<.001	5.622	4.954	6.380
Emphysema (Yes)	0.706	12.35	<.001	4.102	3.273	5.141
Family history of asthma (Yes)	0.431	22.99	<.001	2.370	2.200	2.552

Note: Model 1 was not adjusted; Model 2 adjusted for age, sex, ethnicity, educational level, socioeconomic status, health insurance status, and BMI; Model 3 further adjusted for smoking status, history of diseases (including infection, chronic bronchitis and emphysema), and family history of asthma.

OR: Odds ratio. CI: Confidence interval.

Supplementary Table S2. Subgroup analysis of antibiotics exposure and asthma by interaction of Age and Gender

Variables	*β*	*t*	*p* Value	OR	Lower 95%CI	Upper 95%CI
**Male**						
**20-40**						
Antibiotics (Not Use Antibiotics)	Ref	/	/	/	/	/
Antibiotics (Macrolides derivatives)	0.775	2.17	0.031	1.957	0.877	4.364
Antibiotics (Sulfonamides)	−0.056	−0.06	0.950	0.852	0.114	6.395
Antibiotics (Penicillin)	0.474	1.48	0.142	1.448	0.845	2.481
Antibiotics (Cephalosporins)	0.926	2.16	0.032	2.275	0.970	5.332
Antibiotics (Miscellaneous antibiotics)	−0.589	−1.08	0.284	0.500	0.155	1.614
Antibiotics (Quinolones)	−1.632	−2.24	0.026	0.176	0.031	0.991
**40-60**						
Antibiotics (Not Use Antibiotics)	Ref	/	/	/	/	/
Antibiotics (Macrolides derivatives)	0.772	1.69	0.092	1.918	0.743	4.954
Antibiotics (Sulfonamides)	0.031	0.05	0.958	0.914	0.246	3.402
Antibiotics (Penicillin)	−0.102	−0.27	0.786	0.800	0.373	1.717
Antibiotics (Cephalosporins)	0.103	0.16	0.869	0.983	0.249	3.873
Antibiotics (Miscellaneous antibiotics)	0.069	0.14	0.886	0.949	0.344	2.624
Antibiotics (Quinolones)	−0.994	−1.51	0.134	0.328	0.078	1.384
**>60**						
Antibiotics (Not Use Antibiotics)	Ref	/	/	/	/	/
Antibiotics (Macrolides derivatives)	−0.436	−0.95	0.343	0.843	0.304	2.338
Antibiotics (Sulfonamides)	0.350	0.71	0.476	1.851	0.620	5.523
Antibiotics (Penicillin)	−0.009	−0.02	0.981	1.293	0.603	2.770
Antibiotics (Cephalosporins)	0.445	1.04	0.298	2.036	0.842	4.922
Antibiotics (Miscellaneous antibiotics)	−0.080	−0.22	0.828	1.205	0.558	2.603
Antibiotics (Quinolones)	−0.005	−0.02	0.988	1.298	0.660	2.550
**Female**						
**20-40**						
Antibiotics (Not Use Antibiotics)	Ref	/	/	/	/	/
Antibiotics (Macrolides derivatives)	0.404	0.91	0.365	2.065	0.814	5.243
Antibiotics (Sulfonamides)	−0.558	−1.31	0.191	0.790	0.305	2.048
Antibiotics (Penicillin)	0.034	0.12	0.905	1.427	0.830	2.452
Antibiotics (Cephalosporins)	−0.506	−1.48	0.140	0.832	0.390	1.774
Antibiotics (Miscellaneous antibiotics)	0.067	0.24	0.813	1.475	0.830	2.624
Antibiotics (Quinolones)	0.882	1.84	0.068	3.331	1.131	9.813
**40-60**						
Antibiotics (Not Use Antibiotics)	Ref	/	/	/	/	/
Antibiotics (Macrolides derivatives)	0.915	2.43	0.016	2.302	1.058	5.008
Antibiotics (Sulfonamides)	−1.039	−1.62	0.107	0.326	0.074	1.433
Antibiotics (Penicillin)	0.230	0.73	0.468	1.159	0.631	2.129
Antibiotics (Cephalosporins)	−0.141	−0.34	0.737	0.801	0.322	1.992
Antibiotics (Miscellaneous antibiotics)	−0.391	−1.03	0.303	0.624	0.286	1.360

Antibiotics (Quinolones)	0.343	0.84	0.405	1.299	0.512	3.296
**>60**						
Antibiotics (Not Use Antibiotics)	Ref	/	/	/	/	/
Antibiotics (Macrolides derivatives)	0.399	0.99	0.322	2.134	0.899	5.062
Antibiotics (Sulfonamides)	−0.181	−0.31	0.760	1.194	0.327	4.365
Antibiotics (Penicillin)	−0.071	−0.16	0.874	1.333	0.505	3.522
Antibiotics (Cephalosporins)	−0.762	−1.57	0.118	0.668	0.236	1.889
Antibiotics (Miscellaneous antibiotics)	−0.179	−0.50	0.618	1.197	0.559	2.562
Antibiotics (Quinolones)	1.153	2.22	0.028	4.532	1.482	13.855

OR: Odds ratio. CI: Confidence interval.
